# Role of bacterial pathogens and toxins in sepsis-related myocardial injury: a scoping review

**DOI:** 10.1136/bmjopen-2026-120003

**Published:** 2026-08-26

**Authors:** Samantha Lörstad, Jacob Widaeus, Louise Essehorn, Patrik Gille-Johnson, Jonas Persson

**Affiliations:** 1 Department of Clinical Sciences, Danderyd University Hospital, Division of Internal Medicine, Karolinska Institute, Stockholm, Sweden; 2 Infectious Diseases Clinic, Danderyd University Hospital, Stockholm, Sweden; 3 Department of Clinical Sciences, Danderyd University Hospital, Division of Cardiovascular Medicine, Karolinska Institute, Stockholm, Sweden

**Keywords:** Sepsis, Adult cardiology, BACTERIOLOGY, Review

## Abstract

**Objectives:**

To map and synthesise human and experimental literature examining associations between specific bacterial pathogens or toxins and myocardial injury in adult sepsis and to evaluate whether the available evidence supports pathogen-specific mechanisms of myocardial injury.

**Design:**

Scoping review conducted in accordance with the Joanna Briggs Institute methodology and reported in accordance with the Preferred Reporting Items for Systematic Reviews and Meta-Analyses extension for Scoping Reviews (PRISMA-ScR) guidance.

**Data sources:**

MEDLINE (via PubMed), Embase and Web of Science were searched from inception to December 2024, with an updated search in December 2025. Two reviewers independently screened studies for eligibility and reference lists of relevant articles were searched.

**Eligibility criteria:**

Studies of adults (≥18 years) with sepsis, experimentally induced bacteraemia or experimentally induced endotoxaemia were included if they reported myocardial injury associated with microbiologically identified bacterial pathogens and/or specific toxins. Myocardial injury was defined by circulating cardiac biomarkers and/or histopathological evidence. Animal-only studies were excluded. Only studies published in English were included.

**Data extraction and synthesis:**

Two reviewers independently extracted data using a predefined data extraction form. Findings were synthesised descriptively according to pathogen, study design, methods used to identify myocardial injury and reported cardiac findings.

**Results:**

17 studies met the inclusion criteria, comprising 173 clinical patients from three observational cohort studies and 13 case reports, together with one experimental in vitro study of 23 ex vivo human myocardial tissue samples. A diverse range of Gram-negative and Gram-positive pathogens was reported. Myocardial injury was identified predominantly by circulating biomarkers, with limited postmortem histopathological confirmation. No study was designed to evaluate causal or comparative pathogen-specific mechanisms. Across studies, myocardial injury occurred in heterogeneous infectious contexts without consistent association with specific bacterial species or toxins.

**Conclusion:**

Human evidence linking specific bacterial pathogens or toxins to myocardial injury in sepsis is limited and largely descriptive. Available data do not demonstrate consistent associations between specific bacterial pathogens or toxins and myocardial injury, nor do they permit mechanistic attribution.

**Trial registration number:**

Open Science Framework, https://osf.io/e3pnc/overview.

**Strengths and limitations of this study**

- Reported in accordance with Joanna Briggs Institute methodology and reported following Preferred Reporting Items for Systematic Reviews and Meta-Analyses extension for Scoping Reviews (PRISMA-ScR) guidance, with a pre-published protocol to enhance transparency and reproducibility.
- Comprehensive search of three major databases with no date restrictions, supplemented by manual reference screening.
- Independent dual screening and data extraction using a pre-specified data extraction form.
- Inclusion was limited to English-language publications, which may have resulted in language bias.
- Restriction to published studies reporting species-level pathogen identification and predefined measures of myocardial injury may have excluded partially characterised or unpublished data.

## Introduction

 Sepsis-related myocardial injury is a frequent and clinically important complication of severe infection and is associated with increased morbidity and mortality in adult patients.^1^ Over the past two decades, it has been identified predominantly by elevations in cardiac troponin concentrations, which are independently associated with adverse outcomes.^2–4^ Nevertheless, the underlying biological mechanisms remain incompletely understood. The Fourth Universal Definition of Myocardial Infarction recognises that patients with sepsis may exhibit elevated cardiac troponin values and reductions in ejection fraction, suggesting that these findings may reflect endotoxin-mediated myocardial dysfunction with subsequent recovery following resolution of sepsis.^5^ However, the evidentiary basis from human studies supporting this attribution is not clearly delineated.

The role of specific bacterial pathogens and their associated toxins in sepsis-related myocardial injury has not been systematically investigated in humans. Much of the current mechanistic understanding of sepsis-related myocardial injury is derived from experimentally induced endotoxaemia, typically involving isolated lipopolysaccharide (LPS) exposure in controlled settings and from animal models.^6–8^ In contrast, clinical sepsis represents a dynamic and heterogeneous host-pathogen interaction, characterised by variable microbial burden, diverse pathogen-associated molecular patterns, immune dysregulation, endothelial injury, microcirculatory alterations and concurrent organ dysfunction.^7 9 10^

Experimental studies in cellular systems and animal models provide compelling evidence that bacterial pathogens and their toxins may contribute directly to sepsis-related myocardial injury through several non-ischaemic mechanisms. LPS has been shown to activate Toll-like receptor-dependent inflammatory signalling in cardiomyocytes, resulting in impaired calcium handling, reduced β-adrenergic responsiveness and depressed contractility.^11–15^ Pore-forming exotoxins produced by Gram-positive organisms, including *Staphylococcus aureus* and *Streptococcus pneumoniae*, disrupt cardiomyocyte membranes, leading to calcium overload, mitochondrial dysfunction and myocyte necrosis in vitro and in vivo.^16–19^ Other studies implicate direct cardiomyocyte injury mediated by mitochondrial dysfunction and activation of apoptotic pathways.^20–23^ However, the extent to which these mechanisms translate to human sepsis remains uncertain.

Preliminary searches undertaken during protocol development identified no existing systematic or scoping reviews on this topic.^24^ These searches also showed that the available human literature comprised a small number of methodologically heterogeneous studies, making a scoping review the most appropriate approach to map the available evidence and identify knowledge gaps.

### Aims and objectives

This scoping review aimed to systematically map and synthesise the human and experimental literature examining associations between bacterial pathogens, their toxins and myocardial injury in adult sepsis. We sought to characterise the extent to which pathogen-specific mechanisms of myocardial injury have been investigated in the existing literature and to identify priorities for future clinical and mechanistic research.

## Methods

### Scoping review methodology

This scoping review was conducted in accordance with a pre-published protocol.^24^ The review followed the Joanna Briggs Institute (JBI) methodology and is reported in accordance with the Preferred Reporting Items for Systematic Reviews and Meta-Analyses extension for Scoping Reviews (PRISMA-ScR).^25 26^ The protocol was registered with the Open Science Framework (OSF) on 3 March 2025 (https://osf.io/e3pnc/overview). An initial literature search was conducted in December 2024, with an updated search performed in 2025 prior to manuscript submission. Data supporting the findings of this study are available from the corresponding author on reasonable request.

### Research question

This scoping review addressed the following questions:

1. Which bacterial pathogens and bacterial toxins have been associated with myocardial injury in patients with sepsis?
2. What methods have been used to assess myocardial injury in studies investigating bacterial pathogens or toxins in adult patients with sepsis, including serum cardiac biomarkers, histopathology and cardiac imaging?
3. How was pathogen-associated myocardial injury characterised across studies in terms of structural, microvascular and functional cardiac findings?
4. What study designs have been used to investigate these associations?

### Eligibility criteria

#### Inclusion criteria

This scoping review included studies involving adult human participants (aged ≥18 years) with sepsis, experimentally induced bacteraemia or experimentally induced endotoxaemia. Eligible studies were required to report myocardial injury in the context of sepsis or experimental infection and to identify the causative bacterial pathogen(s) and/or bacterial toxin(s) at the species or molecular level, rather than by broad microbiological classification alone.

Assessment of myocardial injury was required to include circulating cardiac biomarkers and/or histopathological evidence of cardiomyocyte injury. The Fourth Universal Definition of Myocardial Infarction defines myocardial injury on the basis of cardiac troponin elevation.^5^ However, earlier sepsis studies used a broader range of biomarkers, including creatine kinase-MB and myoglobin, before cardiac troponins became the predominant biomarker for defining myocardial injury.^27^ Accordingly, studies were eligible for inclusion if myocardial injury was defined using biomarker-specific definitions and thresholds applied by the original authors.

Clinical studies, including observational studies, interventional studies and experimental endotoxaemia studies, as well as case reports and laboratory studies, were included if they reported myocardial injury and included at least one additional cardiac assessment. Additional cardiac assessments included electrocardiography and cardiac imaging modalities, including echocardiography, coronary angiography and cardiac MRI, with assessment of coronary flow or myocardial perfusion where available. Only studies published in English were eligible for inclusion.

#### Exclusion criteria

Studies were excluded if they were conducted exclusively in animal models. Studies focusing on infective endocarditis, viral myocarditis, aortitis, cardiac abscesses or mycotic aneurysms were excluded. Studies primarily involving patients undergoing pharmacological interventions, extracorporeal therapies (including haemoperfusion or extracorporeal membrane oxygenation), organ transplantation, open-heart surgery or burn-related sepsis were also excluded, as these represent highly selected populations that differ substantially from the broader population of adults with bacterial sepsis.

### Search strategy, study selection and data extraction

A comprehensive literature search was conducted on 12 December 2024 using MEDLINE (via PubMed), Web of Science and Embase. The search strategy was developed around the key concepts of bacterial pathogens and bacterial toxins, myocardial injury and sepsis. The full electronic search strategies for all databases are provided in online supplemental appendix 1 and are reproduced from the published study protocol.^24^

All references were managed using Covidence (2025 Covidence, Melbourne, Australia). Following removal of duplicates, two reviewers (SL and JW) independently screened titles and abstracts for eligibility. Full-text articles were subsequently assessed independently by both reviewers to determine final inclusion. Additional studies were identified through manual screening of reference lists of retrieved articles and relevant review articles addressing sepsis-induced cardiomyopathy.

To ensure inclusion of recently published studies, an updated literature search was performed on 9 December 2025 before manuscript submission.

Data extraction was performed independently by SL and JW using a pre-defined data extraction form developed a priori and adapted from the published study protocol.^24^ One reviewer (SL) compiled the extracted data into a final dataset, which was independently checked by the second reviewer (JW). Any discrepancies were resolved through discussion and consensus. Full extraction fields are presented in online supplemental table S1, while a simplified summary table is shown in the main manuscript for clarity (table 1).

**Table 1:** Characteristics of studies reporting myocardial injury in sepsis with identified pathogens

Study identification	Study design	Population	Bacterial pathogen(s)	Myocardial injury identification	Myocardial injury characterisation	Summary of reported myocardial findings
Monsalve *et al*^28^ (Spain)	Retrospective single-centre cohort	Adults with sepsis/septic shock (n=39)	*Neisseria meningitidis*; other Gram-negative bacilli	CK-MB; autopsy with histopathology (subset)	ECG; M-mode echocardiography	Functional myocardial depression with discordant biomarker, ECG and echocardiographic findings; no consistent histological evidence of myocardial necrosis
Ammann *et al*^29^ (Switzerland)	Prospective single-centre cohort	Adults with sepsis/septic shock (n=20)	Gram-positive, Gram-negative, mixed infections, fungi	CK-MB; troponin I; autopsy (subset)	ECG; echocardiography; coronary angiography	Among troponin-positive patients, Gram-positive organisms predominated, with *Streptococcus pneumoniae* identified in 41% of cases; ECG, imaging and autopsy findings were heterogeneous
Balan *et al*^31^ (UK)	Case report	Adult with septic shock	*Clostridium perfringens*	Troponin I; autopsy with histopathology	Echocardiography; invasive haemodynamics	Biomarker elevation with acute right-ventricular failure and pulmonary vascular dysfunction, without myocardial necrosis
Newton and Sharma^32^ (USA)	Case report	Adult with sepsis	*Capnocytophaga canimorsus*	CK; CK-MB	ECG; echocardiography; coronary angiography	Biomarker elevation with ischaemic-pattern ECG changes and transient coronary thrombosis
Ehrbar *et al*^33^ (Switzerland)	Case report	Adults with sepsis requiring vasopressor support (n=2)	*Capnocytophaga canimorsus*	CK; CK-MB	ECG; echocardiography; coronary angiography	Biomarker elevation with acute ischaemic ECG abnormalities and preserved ventricular systolic function and non-obstructive coronary arteries
Filippatos *et al*^34^ (Greece)	Case report	Adult with septic shock	*Neisseria meningitidis*	CK; CK-MB	Echocardiography; coronary angiography	Biomarker elevation with transient left ventricular systolic dysfunction and regional wall-motion abnormalities with non-obstructive coronary arteries
Gach *et al*^35^ (Belgium)	Case report	Adult with septic shock	*Neisseria meningitidis*	CK; CK-MB	ECG; echocardiography	Biomarker elevation with diffuse ventricular hypokinesia, akinesia of the inferior segments, and ST-segment elevation
Mitchell *et al*^36^ (Australia)	Case report	Adult with septic shock	*Capnocytophaga canimorsus*	CK; troponin I	ECG; echocardiography; coronary angiography	Marked biomarker elevation, transient ECG changes, preserved ventricular systolic function and non-obstructive coronary arteries
Simonson *et al*^37^ (USA)	Case report	Adult with sepsis	*Pseudomonas aeruginosa*	CK; CK-MB; troponin I	Echocardiography; cardiac CT	Biomarker elevation with acute global LV systolic dysfunction and subsequent diffuse myocardial calcification
Martinez *et al*^38^ (USA)	Case report	Adult with septic shock	*Escherichia coli*	CK-MB; troponin I	ECG; echocardiography; coronary angiography	Biomarker elevation with ST-segment elevation and LV systolic dysfunction
van Kruijsdijk *et al*^39^ (Netherlands)	Case report	Adult with septic shock	*Shigella flexneri*	CK-MB; troponin	ECG; cardiac CT	Biomarker elevation with dynamic ECG changes, LV dysfunction and later diffuse myocardial calcification
Yoshihara *et al*^40^ (Japan)	Case report	Adult with septic shock	*Escherichia coli*	CK; CK-MB; troponin I	ECG; echocardiography; cardiac CT	Biomarker elevation with LV systolic dysfunction and progressive extensive myocardial calcification
Caracioni *et al*^41^ (Austria)	Case report	Adult with sepsis	*Staphylococcus aureus*	CK; CK-MB; troponin I	ECG; echocardiography; coronary angiography	Biomarker elevation with transient LV systolic dysfunction (stress-induced cardiomyopathy pattern) and non-obstructive coronary arteries
Garcia-Cardenas *et al*^42^ (Mexico)	Case report	Adult with septic shock	*Escherichia coli*	Troponin I	ECG; SPECT; cardiac CT angiography	Biomarker elevation with perfusion abnormalities, non-obstructive coronary arteries and focal myocardial calcification
Lörstad *et al*^43^ (Sweden)	Case report	Adult with septic shock	*Streptococcus pneumoniae*	hs-cTnT	ECG; echocardiography; coronary angiography	Biomarker elevation with preserved LVEF and reversible coronary microvascular dysfunction
Paraschiv *et al*^30^ (Romania)	Retrospective single-centre cohort	Adults with sepsis/septic shock (n=100)	*Streptococcus* spp.; Enterobacterales; polymicrobial	Troponin	Echocardiography	Reversible left ventricular or biventricular systolic dysfunction reported in a subset of patients
Flesch *et al*^44^ (Germany)	Experimental (in vitro)	Human myocardial tissue (n=23)	Gram-negative endotoxin from *Salmonella typhi*	Histopathology	Myocyte contractility testing	Lower β-adrenergic inotropic responsiveness; no histological evidence of structural, ischaemic or toxic myocardial injury

Cardiac biomarkers and imaging findings were reported as part of routine clinical assessment or descriptive case reporting. No study performed pathogen-stratified hypothesis testing.

CK, creatine kinase; CK-MB, creatine kinase-MB isoenzyme; hs-cTnT, high-sensitivity cardiac troponin T; LPS, lipopolysaccharide; LV, left ventricular; LVEF, left ventricular ejection fraction; SPECT, single-photon emission CT.

### Patient and public involvement

None.

## Results

### Characteristics of included studies

**Figure 1:**
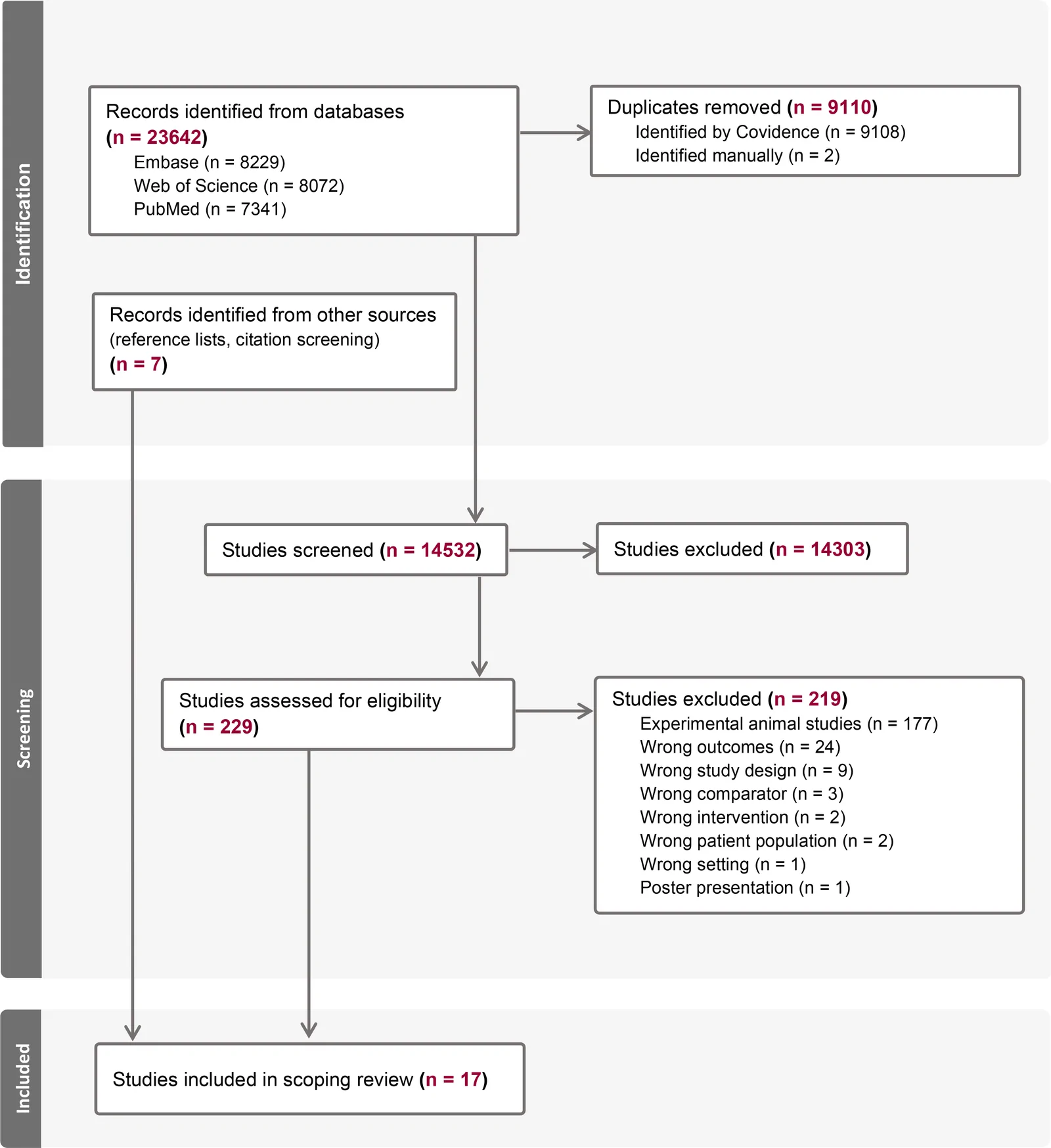
Preferred Reporting Items for Systematic Reviews and Meta-Analyses extension for Scoping Reviews (PRISMA-ScR) flow diagram of the study selection process.

Following screening and eligibility assessment, 17 studies met the inclusion criteria (figure 1). The included studies were published between 1984 and 2025 and represented a heterogeneous and largely descriptive evidence base (table 1). The clinical evidence comprised 173 patients: 159 from three observational single-centre cohort studies^28–30^ and 14 described in 13 case reports.^31–43^ One additional experimental in vitro study investigated endotoxin exposure in 23 ex vivo human myocardial tissue samples.^44^ Detailed extracted data, including serial biomarker values, applied sepsis definitions where reported, and ECG and cardiac imaging findings, are provided in online supplemental table S1.

None of the included studies were designed with the a priori objective of evaluating causal or statistical associations between specific bacterial pathogens and myocardial injury. Instead, cardiac biomarkers and microbiologically identified bacterial pathogens were recorded concurrently in sepsis populations. In cohort studies, cardiac biomarkers were collected systematically but reported descriptively, while in case reports myocardial injury markers were documented as part of routine clinical assessment.

### Reported bacterial pathogens in studies describing myocardial injury

Across the included studies, a diverse range of bacterial pathogens were reported in patients with sepsis and myocardial injury (table 1). These included both Gram-negative organisms (*Escherichia coli*, *Neisseria meningitidis*, *Capnocytophaga canimorsus*, *Pseudomonas aeruginosa* and *Shigella flexneri*) and Gram-positive organisms (*S. pneumoniae*, *S. aureus* and *Clostridium perfringens*). Several studies also reported polymicrobial infections. The experimental study using human myocardial tissue involved incubation in a bathing solution supplemented with LPS (endotoxin) derived from *Salmonella typhi*.^44^

In the clinical studies, bacterial pathogens were identified using routine microbiological investigations, most commonly blood cultures (online supplemental table S1). Pathogen identification was reported alongside markers of myocardial injury. In one prospective cohort, Gram-positive organisms were more frequently observed among troponin-positive patients than Gram-negative organisms, with *S. pneumoniae* identified in 41% of troponin-positive cases.^29^

### Assessment of myocardial injury across included studies

Across the included clinical studies, myocardial injury was identified primarily using circulating cardiac biomarkers. Earlier studies predominantly used creatine kinase-MB isoenzyme (CK-MB), whereas cardiac troponins (cardiac troponin I or high-sensitivity cardiac troponin T) became the predominant biomarkers in studies published after 2000. Where specified, troponin concentrations exceeding the assay reference range were taken as biochemical evidence of myocardial injury.^5^ Biomarker measurements were obtained as part of routine clinical care or study protocols, with substantial variability in assays, thresholds and sampling strategies across studies (online supplemental table S1).

Histopathological assessment was available in only a limited subset of patients and was derived exclusively from postmortem examination.^28 29 31^ Histopathological findings included acute interstitial myocarditis, septic myocarditis, normal myocardial histology and one case of subacute myocardial infarction (online supplemental table S1).

### Characterisation of myocardial injury in studies with identified bacterial pathogens

Across studies with identified bacterial pathogens, myocardial injury was characterised predominantly by circulating biomarkers, with electrocardiography, cardiac imaging, invasive coronary assessment and, in a limited number of cases, histopathological findings providing supplementary information. Non-biomarker cardiac abnormalities were inconsistently reported and varied widely between studies, including transient left ventricular systolic dysfunction, regional wall-motion abnormalities, ischaemic electrocardiographic changes, evidence of coronary microvascular dysfunction and, in rare cases, chronic structural changes such as myocardial calcification (online supplemental table S1). These findings were reported descriptively, without systematic application or standardised definitions, and were not used to define myocardial injury or to support pathogen-specific mechanistic inference.

## Discussion

### Principal findings

This scoping review mapped clinical and experimental evidence relating myocardial injury in adult sepsis to identified bacterial pathogens and toxins. The literature is heterogeneous and largely descriptive, dominated by case reports and small observational cohorts. In clinical studies, myocardial injury was identified primarily using circulating cardiac biomarkers, with histopathology available only in limited postmortem cases. A diverse range of Gram-negative and Gram-positive pathogens was reported, while experimental evidence in humans was confined to direct endotoxin exposure in isolated myocardial tissue. Overall, current human evidence describes myocardial injury across heterogeneous infectious contexts but does not support pathogen-specific or mechanistic inference.

### Interpretation in the context of current definitions of myocardial injury

Myocardial injury in sepsis should be interpreted within contemporary diagnostic frameworks for myocardial injury and infarction. The Fourth Universal Definition of Myocardial Infarction recognises sepsis as a clinical setting in which myocardial injury may occur and emphasises that a diagnosis of myocardial infarction requires evidence of acute myocardial ischaemia in addition to cardiac troponin elevation.^5^ Among studies reporting cardiac troponin concentrations in the present review, troponin elevation generally occurred without evidence of acute coronary occlusion, although isolated cases demonstrated features consistent with myocardial infarction or transient coronary thrombosis.

Myocardial injury and myocardial dysfunction in sepsis should not be regarded as synonymous. Sepsis-induced cardiomyopathy is increasingly recognised as a spectrum of cardiovascular abnormalities, encompassing ventricular dysfunction, myocardial injury, arrhythmias and haemodynamic disturbances.^45^ The mechanisms underlying myocardial injury and dysfunction are likely to be heterogeneous and could plausibly differ according to the causative pathogen. A recent review has proposed that distinct pathogen-specific myocardial dysfunction phenotypes may exist, particularly in streptococcal toxic shock syndrome, although supporting evidence remains limited.^46^

The limited human evidence identified in this review raises an important question: why has endotoxin come to occupy such a central place in contemporary concepts of myocardial injury in sepsis despite the scarcity of comparative human data? The answer likely lies in the historical evolution of the field. Over several decades, a series of clinical observations, physiological studies and experimental endotoxin models have shaped an endotoxin-centred conceptual framework that continues to influence current understanding of myocardial injury in sepsis.^5^

### Evolution of the endotoxin paradigm in sepsis

#### Birth of the endotoxin–shock paradigm

The endotoxin–shock paradigm in sepsis developed through a historical sequence of clinical observation and physiological inference rather than direct mechanistic evidence linking endotoxin to myocardial injury. Early pathological observations, beginning with Brill and Libman’s 1899 description of *Pseudomonas* bacteraemia, were later synthesised by Waisbren (1951) into the concept of Gram-negative sepsis as a toxin-mediated shock syndrome.^47 48^ This framework was subsequently reinforced by haemodynamic studies in the 1960s–1970s demonstrating that patients with Gram-negative bacteraemia exhibited physiological abnormalities closely resembling those observed during experimental endotoxin infusion.^49 50^ The paradigm gained further influence after Suffredini *et al* demonstrated that administration of purified endotoxin to healthy volunteers reproduced key haemodynamic and echocardiographic features of early septic shock, including reversible left ventricular depression, in the absence of bacteraemia.^51^

#### Early challenges to the endotoxin paradigm

Subsequent studies exposed important limitations of an exclusively endotoxin-mediated model of cardiovascular dysfunction. Endotoxaemia was inconsistently detected in human septic shock, myocardial depression was observed across different bacterial infections, and circulating host-derived mediators were shown to depress cardiomyocyte contractility independently of the infecting organism.^52–54^ Nevertheless, these studies remained focused primarily on haemodynamic abnormalities and ventricular dysfunction. Whether endotoxin or other bacterial factors were associated with myocardial injury became a distinct question only with the subsequent adoption of circulating cardiac biomarkers.

#### Biomarker era: distinguishing myocardial injury from myocardial dysfunction

Earlier reliance on CK-MB, a biomarker with limited myocardial specificity in critical illness, constrained the interpretation of myocardial injury in sepsis, whereas the introduction of cardiac troponins into clinical practice in the late 1990s provided a more specific measure of myocardial injury.^55 56^ ver Elst *et al* subsequently demonstrated that elevated cardiac troponins, consistent with myocardial injury, were present in nearly half of patients with early septic shock.^57^ Troponin elevation was strongly associated with left ventricular systolic dysfunction, but did not differ between Gram-negative and Gram-positive infections (this study did not meet the inclusion criteria for the present review).^57^ In a complementary human endotoxaemia model, van Bockel *et al* demonstrated that endotoxin infusion in healthy male volunteers induced marked inflammatory and haemodynamic responses without cardiac troponin release.^58^ Together, these observations marked the beginning of a transition from interpreting cardiac involvement in sepsis primarily through ventricular dysfunction to recognising myocardial injury as a distinct clinical entity.

#### Human pathological evidence

Further insight into the pathological features of myocardial injury in sepsis came from direct human histopathological studies. Schmittinger *et al* performed the first systematic, postmortem histopathological assessment of the myocardium in 20 patients who died from septic shock.^59^ Structural myocardial injury was widespread, comprising diffuse myocytolysis, contraction-band necrosis, inflammatory infiltrates and interstitial oedema. These lesions were distributed throughout the myocardium without apparent regional specificity or association with the infecting pathogen, with Gram-negative, Gram-positive, polymicrobial and fungal infections all represented. Instead, the extent of myocardial injury correlated with catecholamine exposure, supporting a potential contribution of host-mediated stress responses, while no direct relationship with pathogen-specific or endotoxin-mediated toxicity was identified.^59^

### Limitations of experimental models

Experimental endotoxin and live bacterial infection models have been fundamental to mechanistic research in sepsis and have provided important insights into early inflammatory and haemodynamic responses.^6 9^ However, their relevance to myocardial injury in clinical sepsis requires careful qualification. In experimental human studies, endotoxin is administered as a controlled bolus producing rapid transient cytokinaemia, whereas endotoxaemia in clinical sepsis is typically low-grade, heterogeneous, intermittently detectable and lacks a consistent dose–response relationship with disease severity or organ injury.^52^ Likewise, experimental bacterial infection models often employ bacterial burdens and exposure kinetics that differ substantially from human sepsis, even in contemporary models such as caecal ligation and puncture.^60–62^ Consequently, although these models remain valuable for investigating host–pathogen interactions, they do not fully reproduce the prolonged microbial exposure, cumulative systemic stress and evolving host response that characterise human sepsis. This limitation is reflected in current experimental guidelines, which recommend cautious interpretation of findings from endotoxin models.^63^

### Overall interpretation

This scoping review identifies myocardial injury in sepsis as a frequent but non-specific manifestation observed across diverse bacterial infections. Existing human studies do not demonstrate a consistent association between myocardial injury and any particular bacterial pathogen or toxin. The interpretation of myocardial injury in sepsis has been strongly influenced by experimental endotoxin and animal infection models, despite limited comparative human evidence. Current human data remain insufficient to determine whether specific bacterial pathogens or toxins contribute directly to myocardial injury.

### Strengths and limitations

This scoping review was conducted in accordance with JBI methodology and reported in line with PRISMA-ScR guidance. Strengths include a comprehensive, librarian-supported search strategy developed a priori and applied without date restrictions, independent screening by two reviewers and data extraction using a pre-specified framework published in the study protocol, ensuring methodological transparency and reproducibility.

Several limitations should be acknowledged. The evidence base was limited and heterogeneous, comprising predominantly case reports and small observational cohorts, which precluded quantitative synthesis and causal inference. Myocardial injury was identified mainly using circulating cardiac biomarkers collected for clinical rather than research purposes, with substantial variability in assays, thresholds and sampling strategies. In addition, most included studies did not report whether participants had pre-existing cardiomyopathy or other structural heart disease. Accordingly, some findings attributed to sepsis-related myocardial injury may instead have reflected pre-existing cardiac disease. Systematic histopathological assessment was rare and confined to postmortem cases, limiting tissue-level mechanistic insight. None of the included studies were designed specifically to evaluate pathogen-specific mechanisms, and comparisons across bacterial species or toxins were descriptive rather than analytical.

These limitations reflect significant gaps in the existing literature rather than shortcomings of the review methodology and highlight the need for prospective human studies with systematic microbiological characterisation and integrated cardiac phenotyping to elucidate mechanisms of myocardial injury in sepsis.

## Conclusions

Myocardial injury in sepsis is a frequent clinical finding observed across diverse bacterial infections. The available literature is largely descriptive and relies predominantly on circulating cardiac biomarkers, limiting mechanistic conclusions. Although experimental endotoxin and animal models have strongly shaped current concepts, the available human evidence does not permit mechanistic attribution and does not support consistent associations between specific bacterial pathogens or toxins and myocardial injury. Future prospective human studies integrating systematic microbiological characterisation with comprehensive cardiac assessment are needed to clarify the mechanisms underlying myocardial injury in sepsis and determine whether pathogen-specific mechanisms contribute.

## Supplementary material

10.1136/bmjopen-2026-120003 online supplemental appendix 1

10.1136/bmjopen-2026-120003 online supplemental table 1

## Data Availability

Data are available upon reasonable request.
