# Supplementary material for: Role of bacterial pathogens and toxins in sepsis-related myocardial injury: a scoping review
Source: BMJ Open. 2026 Aug 26;16(8):e120003. doi: 10.1136/bmjopen-2026-120003 (PMC13536068; doi:10.1136/bmjopen-2026-120003)
Supplement: Supplementary file 1 [file bmjopen-2026-120003.reviewer_comments.pdf]

PEER REVIEW HISTORY

BMJ Open publishes all reviews undertaken for accepted manuscripts. Reviewers are asked to complete a checklist review form and are provided with free text boxes to elaborate on their assessment. These free text comments are reproduced below.

ARTICLE DETAILS

Title (Provisional)

The role of bacterial pathogens and toxins in sepsis-related myocardial injury: a scoping review

Authors

Lörstad, Samantha; Widaeus, Jacob; Essehorn, Louise; Gille-Johnson, Patrik; Persson, Jonas

---

VERSION 1 - REVIEW

**Reviewer**	**1**
**Name**	**Rozec, Bertrand**
**Affiliation**	**University hospital of Nantes, Anesthesiology and critical care**
**Date**	**29-May-2026**
**COI**	**None**

In this systematic review, conducted using a rigorous methodology and already published in the BMJ, the authors seek to identify which bacteria or bacterial toxins are associated with myocardial injury in patients with septic shock.

Although relatively well written, this systematic review—whose main merit lies in providing a synthesis of the evidence—nevertheless has a number of limitations.

The main limitation lies in the confusion that the concept of myocardial injury may cause in relation to the potential cardiotoxicity of bacteria/bacterial toxins: indeed, it is not necessary to observe myocardial injury to have true cardiotoxicity: certain drugs or toxins, without causing any damage to the integrity of cardiomyocytes or any ischaemia, can lead to myocardial depression. It should also be emphasised that the pathophysiology of cardiovascular damage is highly complex and, as is well known, cannot be reduced to a single mechanism. I believe that for the uninformed reader this is a source of confusion (with a risk of oversimplification such as 'no myocardial injury = no sepsis-related myocardial depression') and that the authors should place greater emphasis on this aspect, particularly in the discussion. On this subject, in 2025, a study examined the complexity of cardiomyopathy in septic shock through a series of Streptococcal Toxic Shock Syndrome

cases, and deserves to be taken into account by the authors (Stevens DL, Bryant AE. Complexities of cardiomyopathy in septic shock. Curr Opin Infect Dis. 1 June 2025;38(3):214–221)

Similarly, I believe a part of discussion on the potential of new biomarkers of heart failure could be of interest (what are the implications of the miRNAs mentioned by Fu X, Yin L. Clinical Study evaluating the Diagnostic Value of Cardiac Ultrasound Parameters Combined With Serum miR-324-3p in Septic Cardiac Dysfunction. APMIS. 2025 Dec;133(12):e70095.

Furthermore, a few points warrant clarification:

- Why were patients undergoing pharmacological interventions or extracorporeal therapies (including haemoperfusion or extracorporeal membrane oxygenation) excluded? Could you explain this choice?

- The total number of patients included is missing

**Reviewer**	**2**
**Name**	**Doyen, Denis**
**Affiliation**	**University Hospital Centre Nice, Médecine Intensive Réanimation**
**Date**	**27-Jun-2026**
**COI**	**None**

The authors present a review protocol aimed at describing the role of bacteria and their toxins in sepsis-related myocardial injury.

The topic is original and particularly challenging, as sepsis-related myocardial injury remains poorly characterized, with heterogeneous definitions and descriptions across the literature.

The review is well written.

One of its strengths is the requirement for structural or functional cardiac abnormalities (echocardiographic findings, electrocardiographic changes, coronary angiography findings, etc.) in association with elevated cardiac biomarkers, which provides a more specific definition of cardiac involvement than an isolated increase in troponin levels.

However, the definition used throughout the review is unclear: does the review focus on sepsis-related myocardial injury or sepsis-related myocardial dysfunction? As cardiac function cannot always be assessed (e.g., systolic or diastolic dysfunction may not be documented), I would suggest consistently using the term *sepsis-related myocardial injury* rather than *sepsis-related myocardial dysfunction*.

Another limitation that should be acknowledged in the discussion section is that a prior history of cardiomyopathy was not described. Patients with pre-existing known

cardiomyopathy should be excluded to ensure that any abnormal cardiologic findings are specific to the sepsis-related myocardial injury and not attributable to another pre-existing condition

---

VERSION 1 - AUTHOR RESPONSE

Reviewer: 1
Prof. Bertrand Rozec, University hospital of Nantes
Comments to the Authors:
In this systematic review, conducted using a rigorous methodology and already published in the BMJ, the authors seek to identify which bacteria or bacterial toxins are associated with myocardial injury in patients with septic shock.

Although relatively well written, this systematic review—whose main merit lies in providing a synthesis of the evidence—nevertheless has a number of limitations.

(1) The main limitation lies in the confusion that the concept of myocardial injury may cause in relation to the potential cardiotoxicity of bacteria/bacterial toxins: indeed, it is not necessary to observe myocardial injury to have true cardiotoxicity: certain drugs or toxins, without causing any damage to the integrity of cardiomyocytes or any ischaemia, can lead to myocardial depression. It should also be emphasised that the pathophysiology of cardiovascular damage is highly complex and, as is well known, cannot be reduced to a single mechanism. I believe that for the uninformed reader this is a source of confusion (with a risk of oversimplification such as 'no myocardial injury = no sepsis-related myocardial depression') and that the authors should place greater emphasis on this aspect, particularly in the discussion. On this subject, in 2025, a study examined the complexity of cardiomyopathy in septic shock through a series of Streptococcal Toxic Shock Syndrome cases, and deserves to be taken into account by the authors (Stevens DL, Bryant AE. Complexities of cardiomyopathy in septic shock. Curr Opin Infect Dis. 1 June 2025;38(3):214–221)

Response (1): We appreciate the Reviewer drawing attention to this issue. We have revised the Discussion section to explicitly distinguish myocardial injury from myocardial dysfunction and to clarify that sepsis-induced cardiomyopathy encompasses a heterogeneous spectrum of cardiac abnormalities. We have also incorporated the suggested reference by Stevens & Bryant (2025) to acknowledge the proposed concept of pathogen-specific cardiovascular phenotypes in sepsis. In addition, we have revised and streamlined the sub-sections discussing endotoxin to place greater emphasis on myocardial injury rather than myocardial dysfunction, thereby improving the alignment of the Discussion with the objectives and scope of the review.

(Page 13, line 20 to Page 17 line 12)

"Discussion:" *Importantly, myocardial injury and myocardial dysfunction in sepsis should not be regarded as synonymous. Sepsis-induced cardiomyopathy is increasingly recognised as a spectrum of cardiovascular abnormalities, encompassing ventricular dysfunction, myocardial injury, arrhythmias, and haemodynamic disturbances.[45] A recent review has proposed that distinct pathogen-specific cardiovascular dysfunction phenotypes may exist, particularly in streptococcal toxic shock syndrome, although supporting evidence remains limited.[46]*

The limited human evidence identified in this review raises an important question: why has endotoxin come to occupy such a central place in contemporary concepts of myocardial injury in sepsis despite the scarcity of comparative human data? The answer lies in the historical evolution of the field. Over several decades, a series of clinical observations, physiological studies, and experimental endotoxin models progressively shaped an endotoxin-centred conceptual framework that continues to influence current understanding of myocardial injury in sepsis.[5]

Evolution of the endotoxin paradigm in sepsis

Birth of the endotoxin–shock paradigm

The endotoxin–shock paradigm in sepsis developed through a historical sequence of clinical observation and physiological inference rather than direct mechanistic evidence linking endotoxin to myocardial injury. Early pathological observations, beginning with Brill and Libman's 1899 description of Pseudomonas bacteraemia, were later synthesised by Waisbren (1951) into the concept of Gram-negative sepsis as a toxin-mediated shock syndrome.[47,48] This framework was subsequently reinforced by haemodynamic studies in the 1960s–1970s demonstrating that patients with Gram-negative bacteraemia exhibited physiological abnormalities closely resembling those observed during experimental endotoxin infusion.[49,50] The paradigm gained further influence after Suffredini et al. (1989) demonstrated that administration of purified endotoxin to healthy volunteers reproduced key haemodynamic and echocardiographic features of early septic shock, including reversible left ventricular depression, in the absence of bacteraemia.[51]

Early challenges to the endotoxin paradigm

Subsequent studies exposed important limitations of an exclusively endotoxin-mediated model of cardiovascular dysfunction. Endotoxaemia was inconsistently detected in human septic shock, myocardial depression was observed across different bacterial infections, and circulating host-derived mediators were shown to depress cardiomyocyte contractility independently of the infecting organism.[52-54] Nevertheless, these studies remained focused primarily on haemodynamic abnormalities and ventricular dysfunction. Whether endotoxin or other bacterial factors were associated with myocardial injury became a distinct question only with the subsequent adoption of circulating cardiac biomarkers.

The biomarker era: distinguishing myocardial injury from myocardial dysfunction

Earlier reliance on CK-MB, a biomarker with limited myocardial specificity in critical illness, constrained the interpretation of myocardial injury in sepsis, whereas the introduction of cardiac troponins into clinical practice in the late 1990s enabled myocardial injury to be defined as a distinct clinical entity.[55,56] Ver Elst et al. (2000) subsequently demonstrated that elevated cardiac troponins, consistent with myocardial injury, were present in nearly half of patients with early septic shock.[57] Troponin elevation was strongly associated with left ventricular systolic dysfunction, but did not differ between Gram-negative and Gram-positive infections (this study did not meet the inclusion criteria for the present review).[57] In a complementary human endotoxaemia model, Van Bockel et al. (2003) demonstrated that endotoxin infusion in healthy male volunteers induced marked inflammatory and haemodynamic responses without cardiac troponin release.[58] Together, these observations marked the beginning of a transition from interpreting cardiac involvement in sepsis primarily through ventricular dysfunction to recognising myocardial injury as a distinct clinical entity.

Human pathological evidence

Further insight into the pathological features of myocardial injury in sepsis came from direct human histopathological studies. Schmittinger et al. (2013) performed the first systematic, post-mortem histopathological assessment of the myocardium in patients who died from septic shock.[59] Structural myocardial injury was ubiquitous, comprising diffuse myocytolysis, contraction-band necrosis, inflammatory infiltrates, and interstitial oedema. These lesions were distributed throughout the myocardium without apparent regional specificity or association with the infecting pathogen, with Gram-negative, Gram-positive, polymicrobial, and fungal infections all represented. Instead, the extent of myocardial injury correlated with catecholamine exposure, supporting a predominant role for host-mediated stress responses rather than direct pathogen-specific or endotoxin-mediated toxicity.[59]

Limitations of experimental models

Experimental endotoxin and live bacterial infection models have been fundamental to mechanistic research in sepsis and have provided important insights into early inflammatory and haemodynamic responses.[6,9] However, their relevance to myocardial injury in clinical sepsis requires careful qualification. In experimental human studies, endotoxin is administered as a controlled bolus producing rapid transient cytokinaemia, whereas endotoxaemia in clinical sepsis is typically low-grade, heterogeneous, intermittently detectable, and lacks a consistent dose–response relationship with disease severity or organ injury.[52] Likewise, experimental bacterial infection models often employ bacterial burdens and exposure kinetics that differ substantially from human sepsis, even in contemporary models such as caecal ligation and puncture.[60-62] Consequently, although these models remain valuable for investigating host–pathogen interactions, they do not fully reproduce the prolonged microbial exposure, cumulative systemic stress, and evolving host response that

characterise human sepsis. This limitation is reflected in current experimental guidelines, which recommend cautious interpretation of findings from endotoxin models.[63]

Overall interpretation

This scoping review identifies myocardial injury in sepsis as a frequent but non-specific manifestation observed across diverse bacterial infections. Existing human studies do not demonstrate a consistent association between myocardial injury and any particular bacterial pathogen or toxin. The interpretation of myocardial injury in sepsis has been strongly influenced by experimental endotoxin and animal infection models, despite limited comparative human evidence. Current human data remain insufficient to determine whether specific bacterial pathogens or toxins contribute directly to myocardial injury."

(2) Similarly, I believe a part of discussion on the potential of new biomarkers of heart failure could be of interest (what are the implications of the miRNAs mentioned by Fu X, Yin L. Clinical Study evaluating the Diagnostic Value of Cardiac Ultrasound Parameters Combined With Serum miR-324-3p in Septic Cardiac Dysfunction. APMIS. 2025 Dec;133(12):e70095.

Response (2): We thank the Reviewer for this suggestion. We carefully considered the study by Fu and Yin (2025). This study provides valuable insights into the diagnosis of sepsis-induced cardiac dysfunction by combining echocardiographic parameters with serum miR-324-3p and includes complementary experimental data exploring mechanisms of cardiomyocyte injury. However, we believe that a detailed discussion of these biomarkers falls outside the predefined scope of the present review, which focuses specifically on the association between bacterial pathogens or toxins and myocardial injury rather than on the diagnosis or pathophysiology of sepsis-induced myocardial dysfunction.

(3) Furthermore, a few points warrant clarification:
- Why were patients undergoing pharmacological interventions or extracorporeal therapies (including haemoperfusion or extracorporeal membrane oxygenation) excluded? Could you explain this choice?

Response (3): We agree that the rationale for this exclusion criterion required clarification. We have revised the Methods section to explain that studies involving pharmacological interventions, extracorporeal therapies (including haemoperfusion and extracorporeal membrane oxygenation), organ transplantation, open-heart surgery, and burn-related sepsis were excluded because these highly selected populations differ substantially from the broader population of patients with bacterial sepsis,

thereby limiting comparability across the included studies. This clarification has been added to the Exclusion criteria subsection.

Methods (Exclusion criteria) (Page 8,line 23 to Page 9, line 3): "*Studies primarily involving patients undergoing pharmacological interventions, extracorporeal therapies (including haemoperfusion or extracorporeal membrane oxygenation), organ transplantation, open-heart surgery, or burn-related sepsis were also excluded, as these represent highly selected populations that differ substantially from the broader population of adults with bacterial sepsis*."

(4) - The total number of patients included is missing

Response (4): We thank the Reviewer for highlighting this omission. We have now reported the total number of included clinical patients in both the Abstract and the *Characteristics of included studies* section of the main text. To distinguish the clinical evidence from the experimental study, we also specify that the latter comprised 23 *ex vivo* human myocardial tissue samples.

Abstract (Page 2, line 22 to Page 3, line 1) "*Results Seventeen studies met the inclusion criteria, comprising 173 clinical patients from three observational cohort studies and 13 case reports, together with one experimental in vitro study of 23 ex vivo human myocardial tissue samples*."

Methods (Characteristics of included studies) (Page 10, Lines 9-16): "*Following screening and eligibility assessment, 17 studies met the inclusion criteria (Figure 1). The included studies were published between 1984 and 2025 and represented a heterogeneous and largely descriptive evidence base (Table 1). The clinical evidence comprised 173 patients: 159 from three observational single-centre cohort studies[25-27] and 14 described in 13 case reports.[28-40] One additional experimental in vitro study investigated endotoxin exposure in 23 ex vivo human myocardial tissue samples.[41] Detailed extracted data, including serial biomarker values, applied sepsis definitions where reported, and electrocardiogram and cardiac imaging findings, are provided in Supplementary Table S1*."

****

Reviewer: 2
Dr. Denis Doyen, University Hospital Centre Nice
Comments to the Authors:
The authors present a review protocol aimed at describing the role of bacteria and their toxins in sepsis-related myocardial injury.

The topic is original and particularly challenging, as sepsis-related myocardial injury remains poorly characterized, with heterogeneous definitions and descriptions across the literature.
The review is well written.

One of its strengths is the requirement for structural or functional cardiac abnormalities (echocardiographic findings, electrocardiographic changes, coronary angiography findings, etc.) in association with elevated cardiac biomarkers, which provides a more specific definition of cardiac involvement than an isolated increase in troponin levels.

(1) However, the definition used throughout the review is unclear: does the review focus on sepsis-related myocardial injury or sepsis-related myocardial dysfunction? As cardiac function cannot always be assessed (e.g., systolic or diastolic dysfunction may not be documented), I would suggest consistently using the term *sepsis-related myocardial injury* rather than *sepsis-related myocardial dysfunction*.

Response (1): We thank the Reviewer for highlighting this issue. We agree that the terminology required clarification. Throughout the revised manuscript, we have consistently focused on sepsis-related myocardial injury and removed or revised wording that could be interpreted as equating myocardial injury with myocardial dysfunction. References to sepsis-induced cardiomyopathy have been retained only where necessary to provide clinical context or to describe the literature search strategy. We have also added a clarifying paragraph to the Discussion to explicitly distinguish myocardial injury from myocardial dysfunction. In addition, we have revised and streamlined the subsections discussing endotoxin to place greater emphasis on myocardial injury rather than myocardial dysfunction, thereby improving the alignment of the Discussion with the objectives and scope of the review (pages 13–17; see revised text inserted under Reviewer 1, Response (1)).

Abstract: removed Key Word "*Sepsis-associated myocardial dysfunction*" (Page 3, Lines 13-14)

Introduction: This part of the sentence has been removed "~~*and it remains uncertain whether myocardial involvement in adult sepsis represents transient, reversible dysfunction or direct myocardial injury*~~." (Page 5, lines 11-13 in the originally submitted manuscript)

Introduction: "*Much of the current mechanistic understanding of sepsis-related myocardial ~~dysfunction~~ injury is derived from experimentally induced endotoxaemia…*" (Page 5, lines 13-15).

Methods: "*Additional studies were identified through manual screening of reference lists from retrieved articles and relevant review articles addressing ~~sepsis-related myocardial dysfunction~~ sepsis-induced cardiomyopathy*." (Page 9, lines 14-16)

Discussion (Page 13, line 20 to Page 14, line 2): " *Importantly, myocardial injury and myocardial dysfunction in sepsis should not be regarded as synonymous. Sepsis-induced cardiomyopathy is increasingly recognised as a spectrum of cardiovascular abnormalities, encompassing ventricular dysfunction, myocardial injury, arrhythmias, and haemodynamic disturbances.*[45] *A recent review has proposed that distinct pathogen-specific cardiovascular dysfunction phenotypes may exist, particularly in streptococcal toxic shock syndrome, although supporting evidence remains limited.*[46]"

(2) Another limitation that should be acknowledged in the discussion section is that a prior history of cardiomyopathy was not described. Patients with pre-existing known cardiomyopathy should be excluded to ensure that any abnormal cardiologic findings are specific to the sepsis-related myocardial injury and not attributable to another pre-existing condition.

Response: We agree that the absence of information regarding pre-existing cardiomyopathy or other structural heart disease represents an important limitation when interpreting cardiac abnormalities observed during sepsis. We have therefore revised the Limitations section to acknowledge that most included studies did not report baseline cardiac disease and that, consequently, some findings attributed to sepsis-related myocardial injury may instead have reflected pre-existing cardiac disease.

Discussion (Page 18 lines 1-4) : "*In addition, most included studies did not report whether participants had pre-existing cardiomyopathy or other structural heart disease. Accordingly, some findings attributed to sepsis-related myocardial injury may instead have reflected pre-existing cardiac disease*."

****

Authors's addition: In addition to the revisions made in response to the reviewers' comments, we made minor editorial revisions throughout the manuscript to improve clarity, readability, and flow. We also streamlined the *Introduction* and *Aims and Objectives* sections to reduce repetition and to more clearly explain the rationale for the scoping review based on the preliminary searches conducted during protocol development. These revisions do not alter the scientific content, interpretation, or conclusions of the manuscript and are documented in the tracked-changes version.

Introduction (Page 6, line 3-18):

..... “*Other studies implicate direct cardiomyocyte injury mediated by mitochondrial dysfunction and activation of apoptotic pathways.*[19-22] *However, the extent to which these mechanisms translate to human sepsis remains uncertain.*

Preliminary searches undertaken during protocol development identified no existing systematic or scoping reviews on this topic.[23] *These searches also showed that the available human literature comprised a small number of methodologically heterogeneous studies, making a scoping review the most appropriate approach to map the available evidence and identify knowledge gaps*.

Aims and Objectives

This scoping review aimed to systematically map and synthesise the human and experimental literature examining associations between bacterial pathogens, their toxins, and myocardial injury in adult sepsis. We sought to characterise the extent to which pathogen-specific mechanisms of myocardial injury have been investigated in the existing literature and to identify priorities for future clinical and mechanistic research.”

VERSION 2 - REVIEW

Reviewer 1

Name Rozec, Bertrand

Affiliation University hospital of Nantes, Anesthesiology and critical care

Date 12-Aug-2026

COI

Thank you to the authors for their efforts to clarify the message. I have no further comments.

Reviewer 2

Name Doyen, Denis

Affiliation University Hospital Centre Nice, Médecine Intensive Réanimation

Date 13-Aug-2026

COI

The authors have satisfactorily addressed the comments.
