# Supplementary material for: Role of bacterial pathogens and toxins in sepsis-related myocardial injury: a scoping review
Source: BMJ Open. 2026 Aug 26;16(8):e120003. doi: 10.1136/bmjopen-2026-120003 (PMC13536068; doi:10.1136/bmjopen-2026-120003)
Supplement: Supplementary file 2 [file bmjopen-16-8-s001.docx]

**Supplementary Appendix 1**

**(i) Database searching: MEDLINE (PubMed)**

**Search strategy**

(Bacteria[MeSH Terms] OR “Bacterial Toxins”[MeSH Terms] OR bacteria*[Title/Abstract] OR "Gram-Negative"[Title/Abstract] OR "Gram-Positive"[Title/Abstract] OR Toxin*[Title/Abstract] OR Endotoxin*[Title/Abstract] OR LPS[Title/Abstract] OR Lipopolysaccharide*[Title/Abstract] OR “Lipoteichoic acid*”[Title/Abstract] OR Streptococcus[Title/Abstract] OR “Escherichia coli”[Title/Abstract] OR “E Coli”[Title/Abstract] OR staphylococcus[Title/Abstract] OR Exotoxin[Title/Abstract] OR Enterotoxin[Title/Abstract])

**AND**

(Sepsis[MeSH Terms] OR Bacteremia[MeSH Terms] OR Endotoxemia[MeSH Terms] OR "Shock, Septic"[MeSH Terms] OR “Systemic Inflammatory Response Syndrome”[MeSH Terms] OR Sepsis[Title/Abstract] OR “Bloodstream infection*”[Title/Abstract] OR Bacteremia[Title/Abstract] OR Bacteraemia[Title/Abstract] OR Septicemia[Title/Abstract] OR Septicaemia[Title/Abstract] OR Endotoxemia[Title/Abstract] OR Endotoxaemia[Title/Abstract] OR Septic[Title/Abstract] OR “Septic Shock”[Title/Abstract] OR “Systemic Inflammatory Response Syndrome”[Title/Abstract] OR SIRS[Title/Abstract] OR “Systemic infection*” [Title/Abstract])

**AND**

(Myocardium[MeSH Terms] OR Cardiomyopathies[MeSH Terms] OR Troponin[MeSH Terms] OR “Creatine Kinase”[MeSH Terms] OR “Natriuretic peptides”[MeSH Terms] OR “Cardiac Imaging Techniques”[MeSH Terms] OR cardio*[Title/Abstract] OR myocard*[Title/Abstract] OR cardiac*[Title/Abstract] OR cardial*[Title/Abstract] OR “Contractile dysfunction”[Title/Abstract] OR “Ventricular dysfunction”[Title/Abstract])

**(ii) Database searching: Web of Science**

**Search strategy**

Bacteria* OR “Bacterial toxin” OR Toxin* OR “Gram-negative” OR “Gram-positive” OR Endotoxin* OR LPS OR Lipopolysaccharide* OR “Lipoteichoic acid” OR Exotoxin* OR Streptococcus OR staphylococcus OR “Escherichia coli” OR “E Coli”

**AND**

Sepsis* OR Bacteremia OR Endotoxemia OR Endotoxaemia OR Septicemia OR Bacteraemia OR Septicaemia OR “Bloodstream infection*” OR “Septic Shock” OR “Systemic Inflammatory Response Syndrome” OR SIRS OR “Systemic infection”

**AND**

Cardiomyopath* OR Troponin OR “Creatine Kinase” OR “Natriuretic peptide*” OR “Cardiac Imaging” OR cardio* OR myocard* OR cardiac* OR cardial* OR “Contractile dysfunction” OR “Ventricular dysfunction”

**(iii) Database searching: Embase**

**Search strategy**

(Bacterium/exp OR 'Bacterial Toxin’/exp OR bacteria*:ti,ab OR Gram-Negative:ti,ab OR Gram-Positive:ti,ab OR Toxin*:ti,ab OR Endotoxin*:ti,ab OR LPS:ti,ab OR Lipopolysaccharide*:ti,ab OR 'Lipoteichoic acid*':ti,ab OR Streptococcus:ti,ab OR 'Escherichia coli':ti,ab OR 'E Coli':ti,ab OR staphylococcus:ti,ab OR Exotoxin:ti,ab OR Enterotoxin:ti,ab)

**AND**

(Sepsis/exp OR Bacteremia/exp OR Endotoxemia/exp OR 'Septic shock'/exp OR 'Systemic Inflammatory Response Syndrome'/exp OR

Sepsis*:ti,ab OR 'Bloodstream infection*':ti,ab OR Bacteremia:ti,ab OR Bacteraemia:ti,ab OR Septicemia:ti,ab OR Septicaemia:ti,ab OR Endotoxemia:ti,ab OR Endotoxaemia:ti,ab OR Septic:ti,ab OR 'Septic Shock':ti,ab OR 'Systemic Inflammatory Response Syndrome':ti,ab OR SIRS:ti,ab OR 'Systemic infection*':ti,ab)

**AND**

(Myocardium/exp OR Cardiomyopathy/exp OR Troponin/exp OR 'Creatine Kinase'/exp OR 'Natriuretic factor'/exp OR 'Cardiac imaging'/exp OR cardio*:ti,ab OR myocard*:ti,ab OR cardiac*:ti,ab OR cardial*:ti,ab OR 'Contractile dysfunction':ti,ab OR 'Ventricular dysfunction':ti,ab)
