# Supplementary material for: Role of bacterial pathogens and toxins in sepsis-related myocardial injury: a scoping review
Source: BMJ Open. 2026 Aug 26;16(8):e120003. doi: 10.1136/bmjopen-2026-120003 (PMC13536068; doi:10.1136/bmjopen-2026-120003)
Supplement: Supplementary file 3 [file bmjopen-16-8-s002.docx]

**Supplementary Table S1. Detailed data extraction from included studies**

| **Study identification** | **Study design** | **Population, model** | **Bacterial pathogen** | **Histopathology** | **Biomarkers used to assess myocardial injury** | **Structural/functional cardiac assessment** | **Key myocardial findings** | **Clinical outcomes (if reported)** |
| --- | --- | --- | --- | --- | --- | --- | --- | --- |
| Monsalve et al. (1984);^1^ Spain | Retrospective observational single-centre cohort study | Adults with sepsis or septic shock; sepsis criteria not specified; n=39 | Group M: *Neisseria meningitidis* (n=19)  Group S: Other Gram-negative bacilli (*Escherichia coli, Pseudomonas, Proteus, Enterobacter, Serratia*) (n=20) (blood cultures) | Autopsy performed in three patients demonstrated acute interstitial myocarditis; intracellular Gram-negative diplococci identified in myocardial tissue from one patient | CK-MB elevation was reported in patients with echocardiographic myocardial dysfunction and ECG abnormalities, without reported quantitative values. | Sequential ECG and M-mode echocardiography were normal in 29 patients; impaired myocardial contractility was observed in patients with CK-MB elevation, while three patients had ECG abnormalities despite normal CK-MB, two with pericardial effusion. | Functional myocardial depression with discordant biomarker, ECG, and echocardiographic findings, without consistent evidence of myocardial necrosis; pathogen-specific enzyme associations not assessed | 17/39 patients survived (9/19 meningococcal; 8/20 other Gram-negative bacteraemia) |
| Ammann et al. (2001);^2^ Switzerland | Prospective observational single-centre cohort study | Adults with sepsis, SIRS, or septic shock diagnosed according to ACCP/SCCM 1992 criteria; n=20 | Gram-negative (n=8), Gram-positive (n=4), mixed bacterial infections (n=2), fungal (n=2), and unidentified infections (n=4). Identified bacterial pathogens included *Klebsiella pneumoniae, Proteus mirabilis, Acinetobacter baumannii, Haemophilus parainfluenzae, Escherichia coli, Streptococcus pneumoniae, Streptococcus pyogenes, Staphylococcus aureus, and Legionella pneumophila (blood and other clinical cultures*) | Autopsy performed in five patients: three showed normal myocardial findings; one demonstrated septic myocarditis; one had two-vessel coronary artery disease with subacute apical myocardial infarction | Cardiac troponin I (reference <0.1 ng/mL) was elevated in 17/20 patients (85%) (median 0.57 µg/L, range 0–15.4); CK-MB levels were higher in troponin-positive than troponin-negative patients (33 vs 25 U/L), while controls remained within the normal range | No patients had symptoms or clinical signs of manifest coronary artery disease; non-diagnostic ST–T-segment changes (<1 mm) occurred in 9 troponin-positive and 2 troponin-negative patients; echocardiography was performed in 11 patients, coronary angiography in 1, and autopsy in 5, demonstrating heterogeneous findings including normal coronary arteries in some troponin-positive patients | Troponin-defined myocardial injury occurring across Gram-positive and Gram-negative infections. Gram-positive organisms, particularly *Streptococcus pneumoniae*, were more frequently reported among troponin-positive patients, although no formal pathogen-based comparison was performed. | Six patients died during hospitalisation; five of these had elevated cardiac troponin I levels |
| Balan et al. (2017);^3^ United Kingdom | Case report | Adult with septic shock; sepsis criteria not specified; n=1 | *Clostridium perfringens* (blood culture) | Hospital post-mortem revealed patent coronary arteries, no pulmonary emboli, and isolated marked right ventricular dilatation with normal myocardial histology; no hepatic pathology and no identifiable surgical source of infection | Troponin I markedly elevated at 9.11 ng/mL (ref <0.01 ng/mL) | Normal admission ECG; echocardiography and invasive haemodynamics demonstrated acute RV failure with pulmonary hypertension and elevated pulmonary vascular resistance, consistent with acute cor pulmonale. | Troponin-defined myocardial injury with acute RV failure and pulmonary vascular dysfunction, in the absence of structural myocardial necrosis | Died within 24 hours of admission |
| Newton et al. (1986);^4^ United States | Case report | Adult with sepsis; sepsis criteria not specified; n=1 | *Capnocytophaga canimorsus* (blood culture) | Not reported | Marked elevations of CK (peak 2,130 U/L; reference 22–269 U/L) with elevated CK-MB fraction (18.1%), followed by progressive decline over four days | ECG evidence of anterolateral myocardial infarction with preserved systolic function on echocardiography and transient coronary thrombosis with partial recanalisation on angiography | Enzyme-defined myocardial infarction associated with transient coronary thrombosis during sepsis, with preserved ventricular systolic function | Recovered and discharged after 43 days |
| Ehrbar et al. (1996);^5^ Switzerland | Case report | Adult with sepsis (author-defined) requiring vasopressor support; sepsis criteria not specified; n=2 | *Capnocytophaga canimorsus* (blood culture) | Not reported | Patient 1: CK 3,729 U/L (reference 10–190 U/L) with CK-MB 406 U/L (reference <27 U/L); Patient 2: CK increased from 481 to 1,539 U/L (reference 5–270 U/L) with CK-MB peak 122 U/L (reference <30 U/L) | Both patients demonstrated acute ischaemic ECG abnormalities with preserved LV systolic function on TTE and unobstructed coronary arteries on angiography | Enzyme-defined myocardial injury with acute ischaemic ECG abnormalities in the absence of echocardiographic dysfunction or obstructive CAD | Both patients recovered and were discharged |
| Filippatos et al. (2000);^6^ Greece | Case report | Adult with septic shock; sepsis criteria not specified; n=1 | *Neisseria meningitidis* (blood culture) | Not reported | Marked elevations of CK (2,000 U/L) and CK-MB (373 U/L); reference ranges not reported | Acute LV systolic dysfunction (LVEF 30%) with inferior wall hypokinesia during septic shock, followed by recovery of systolic function and unobstructed coronary arteries on follow-up angiography | Enzyme-defined myocardial injury with acute LV systolic dysfunction and regional wall-motion abnormalities in the absence of obstructive coronary artery disease | Recovered and discharged after two weeks |
| Gach et al. (2001);^7^ Belgium | Case report | Adult with septic shock; sepsis criteria not specified; n=1 | *Neisseria meningitidis* (confirmed from cerebrospinal fluid) | Not reported | Serial CK and CK-MB showed a marked rise–fall pattern (CK peak 3,454 IU/L; CK-MB peak 261.9 µg/L), consistent with acute myocardial injury; reference ranges were not reported. | Admission ECG demonstrated convex upward ST-segment elevation in inferior and lateral leads; TTE showed diffuse ventricular hypokinesia with akinesia of the inferior segments and no pericardial effusion | Enzyme-defined myocardial injury with concurrent electrocardiographic and echocardiographic abnormalities | Discharged in good clinical condition after 28 days |
| Mitchell et al. (2002);^8^ Australia | Case report | Adult with septic shock; sepsis criteria not specified; n=1 | *Capnocytophaga canimorsus* (blood culture) | Not reported | Marked elevations of CK (peak 1,975 IU/L; reference 20‒200 U/L) and cardiac troponin I (peak 148.0 µg/L; reference <0.2 µg/L), peaking within the first 48 hours of ICU admission during reversal of shock and coagulopathy | Transient ECG abnormalities with normal LV function on TTE, preserved cardiac index under vasoactive support, and unobstructed coronary arteries on angiography | Biomarker-defined myocardial injury with marked biomarker elevation in the absence of echocardiographic dysfunction or obstructive CAD | Recovered and discharged following sepsis |
| Simonson et al. (2007);^9^ United States | Case report | Adult with severe sepsis (author-defined) requiring vasopressor support; sepsis criteria not specified; n=1 | *Pseudomonas aeruginosa* (blood, urine and tracheal culture) | Not reported | Marked elevations of CK peak ~4,000 U/L (normal 20–315 U/L), CK-MB 402 ng/mL (normal <5 ng/mL), and troponin I >50 ng/mL (normal <2 ng/mL) | Sepsis-associated acute global LV systolic dysfunction (LVEF 20%) with subsequent functional improvement (LVEF 55%), followed by development of persistent diffuse LV myocardial calcification on serial CT imaging. | Biomarker-defined myocardial injury with acute systolic dysfunction and subsequent development of persistent diffuse myocardial calcification. | Recovered and discharged following sepsis |
| Martinez et al. (2009);^10^ United States | Case report | Adult with septic shock; sepsis criteria not specified; n=1 | *Escherichia coli* (blood and urine cultures) | Not reported | CK-MB 8.6 U/L (reference range not reported); Troponin I 9.6 ng/mL (reference <1.0 ng/mL) | ECG: ST-segment elevation in V1–V2; TTE: LVEF 35% with regional wall-motion abnormalities and diastolic dysfunction; Coronary angiography: normal coronary arteries | Biomarker-defined myocardial injury with ventricular dysfunction in the absence of obstructive CAD | Discharged from ICU in stable condition |
| van Kruijsdijk et al. (2011);^11^ Netherlands | Case report | Adult with septic shock; sepsis criteria not specified; n=1 | *Shigella flexneri* (blood and faecal cultures) | Not reported | Elevated CK-MB (peak 117 ng/mL) and troponin (peak 5.72 ng/mL); troponin assay and reference ranges not reported | Transient ECG abnormalities (diffuse ST-segment elevation and PR depression) with subsequent LV dysfunction and later development of diffuse LV myocardial calcification on imaging | Biomarker-defined myocardial injury with dynamic ECG changes, LV dysfunction, and subsequent diffuse myocardial calcification | Not reported |
| Yoshihara et al. (2022);^12^ Japan | Case report | Adult with septic shock; sepsis criteria not specified; n=1 | *Escherichia coli* (blood culture) | Not reported | Marked elevations of CK (peak 1,378 U/L; reference 56–244 U/L), CK-MB (15.8 ng/mL; reference <4.0 ng/mL), and troponin I (47.3 ng/mL; reference <0.04 ng/mL), with prolonged troponin elevation to day 16 | ECG abnormalities with poor R-wave progression; TTE showed LV systolic dysfunction (LVEF 41%) with progressive diffuse LV myocardial calcification on cardiac CT (day 63) | Severe biomarker-defined myocardial injury with LV dysfunction and progressive diffuse myocardial calcification | Survived to hospital discharge (day 97) |
| Caracioni et al. (2025);^13^ Austria | Case report | Adult with sepsis and cardiogenic shock; SIRS criteria applied; n=1 | *Staphylococcus aureus* (blood culture) | Not reported | Elevated cardiac biomarkers with troponin I rising from 2.67 to 10.14 ng/mL, total CK 1,063 U/L, and CK-MB 22.9 ng/mL; reference ranges not reported | ECG showed mild ST-segment abnormalities; echocardiography demonstrated reduced LV systolic function with basal–mid-ventricular hypokinesia and an apical hyperdynamic region, with subsequent global hypokinesia on follow-up; coronary angiography excluded obstructive CAD | Troponin-defined myocardial injury with stress-induced LV systolic dysfunction consistent with reverse Takotsubo cardiomyopathy, in the absence of obstructive CAD | Recovered from sepsis and Takotsubo cardiomyopathy; discharged around day 25 |
| Garcia-Cardenas et al. (2023);^14^ Mexico | Case report | Adult with septic shock; sepsis criteria not specified; n=1 | *Escherichia coli* (blood culture) | Not reported | Troponin I elevated on day 1 (11.2 ng/mL; reference ≤0.04 ng/mL) with normalisation by day 5 (0.02 ng/mL) | Normal ECG and POCUS during admission; subsequent SPECT imaging demonstrated inferolateral perfusion defects with reverse redistribution, and cardiac CT angiography showed non-obstructive coronary arteries and focal LV lateral wall myocardial calcification | Troponin-defined myocardial injury with biomarker normalisation, non-obstructive coronary arteries, perfusion abnormalities, and focal myocardial calcification | Recovered from sepsis |
| Lörstad et al. (2025);^15^ Sweden | Case report | Adult with septic shock; sepsis criteria not specified; n=1 | *Streptococcus pneumoniae* (blood culture) | Not reported | hs-cTnT rose from 24 to 43 ng/L before declining to 19 ng/L (reference <15 ng/L) | Normal ECG; TTE showed preserved LVEF with reduced stroke volume, impaired global longitudinal strain, and left ventricular–arterial uncoupling; invasive coronary assessment demonstrated severe but reversible coronary microvascular dysfunction, unmasking functionally significant LAD disease on follow-up | Troponin-defined myocardial injury with preserved LVEF and reversible sepsis-related coronary microvascular dysfunction, possible leading to underestimation of epicardial stenosis severity during septic shock | Asymptomatic at 1-month post-PCI follow-up with no reported cardiovascular events |
| Paraschiv et al. (2025);^16^ Romania | Retrospective observational single-centre cohort study | Adults with sepsis or septic shock; Sepsis-3 criteria; culture-positive sepsis; n=100. | Streptococcus spp. (*Streptococcus pneumoniae*, *S. viridans*, *S. pyogenes*); Enterobacterales (*Escherichia coli*, *Proteus mirabilis*) and polymicrobial infections (blood and other clinical cultures) | Not reported | Troponin levels were higher in SICD than non-SICD patients (median 0.68 [IQR 0.0–2.7] vs 0.10 [0.0–0.29] ng/mL; reference ≤0.12 ng/mL), but differences were not statistically significant (p = 0.097) | TTE assessed left and right ventricular systolic function (LVEF, TAPSE); sepsis-induced cardiac dysfunction (SICD) was defined as new reversible left or biventricular systolic dysfunction over 7‒10 days and was identified in 14% of patients. | Streptococcal infections and polymicrobial cultures were associated with SICD. Enterobacterales were more frequent in the SICD group but were not significantly associated with SICD. No patients with SICD had *Staphylococcus* spp. infection. Associations between individual bacterial pathogens and troponin elevation were not assessed | 44% of patients died during hospitalisation, with no difference between SICD and non-SICD patients |
| Flesch et al. (1999);^17^ Germany | Controlled laboratory experimental study | Human myocardial tissue from transplant and donor hearts (n=23; failing n=18, nonfailing n=5); no infection or sepsis; endotoxin exposure applied *in vitro* | Gram-negative endotoxin (LPS) derived from *Salmonella typhi* | Haematoxylin–eosin and Luxol fast blue staining showed no morphological evidence of acute ischaemic or toxic myocardial injury, regardless of endotoxin exposure. | No circulating or tissue cardiac injury biomarkers measured | Isometric force of contraction was measured in electrically stimulated human myocardial tissue. Prolonged endotoxin exposure markedly reduced β-adrenergic (isoprenaline-induced) inotropic responsiveness in both failing and non-failing myocardium. | Endotoxin-associated myocardial depression with inducible nitric oxide synthase expression and attenuation of contractile dysfunction by nitric oxide synthase inhibition and radical scavenging, implicating nitric oxide–dependent pathways | Not applicable |
| **Abbreviations:** ACCP/SCCM 1992 criteria = American College of Chest Physicians/Society of Critical Care Medicine 1992 consensus criteria; CAD = coronary artery disease; CK = creatine kinase; CK-MB = creatine kinase–MB isoenzyme; CT = computed tomography; ECG = electrocardiogram; hs-cTnT = high-sensitivity cardiac troponin T; LPS = lipopolysaccharide; LV = left ventricular; LVEF = left ventricular ejection fraction; PCI = percutaneous coronary intervention; RV = right ventricular; SIRS = systemic inflammatory response syndrome; SPECT = single-photon emission computed tomography; TTE = transthoracic echocardiography. | | | | | | | | |

**References**

1.	Monsalve F, Rucabado L, Salvador A, Bonastre J, Cuñat J, Ruano M. Myocardial depression in septic shock caused by meningococcal infection. *Crit Care Med*. Dec 1984;12(12):1021-3. doi:10.1097/00003246-198412000-00003

2.	Ammann P, Fehr T, Minder EI, Günter C, Bertel O. Elevation of troponin I in sepsis and septic shock. *Intensive Care Med*. Jun 2001;27(6):965-9. doi:10.1007/s001340100920

3.	Balan C, Barker G, Garry D. Clostridium perfringens sepsis complicated by right ventricular cardiogenic shock. *J Intensive Care Soc*. May 2017;18(2):130-137. doi:10.1177/1751143716683713

4.	Newton NL, Sharma B. Acute myocardial infarction associated with DF-2 bacteremia after a dog bite. *Am J Med Sci*. May 1986;291(5):352-4. doi:10.1097/00000441-198605000-00012

5.	Ehrbar HU, Gubler J, Harbarth S, Hirschel B. Capnocytophaga canimorsus sepsis complicated by myocardial infarction in two patients with normal coronary arteries. *Clin Infect Dis*. Aug 1996;23(2):335-6. doi:10.1093/clinids/23.2.335

6.	Filippatos G, Kardara D, Paramithiotou E, et al. Acute myocardial infarction with normal coronary arteries during septic shock from Neisseria meningitidis. *Intensive Care Med*. Feb 2000;26(2):252. doi:10.1007/s001340050058

7.	Gach O, Lancellotti P, Pierard LA. Acute ST-segment elevation in Neisseria meningitis. *Acta Cardiol*. Oct 2001;56(5):327-9. doi:10.2143/ac.56.5.2005695

8.	Mitchell I, McNeillis N, Bowden FJ, Nikolic G. Electrocardiographic myocardial infarction pattern in overwhelming post-splenectomy sepsis due to Capnocytophaga canimorsus. *Intern Med J*. Aug 2002;32(8):415-8. doi:10.1046/j.1445-5994.2002.00236.x

9.	Simonson S, Miller WT, Jr., Perl A, Torigian DA. Diffuse left ventricular myocardial calcification in the setting of sepsis on CT imaging. *J Thorac Imaging*. Nov 2007;22(4):343-5. doi:10.1097/RTI.0b013e31806ad289

10.	Martinez JD, Babu RV, Sharma G. Escherichia coli septic shock masquerading as ST-segment elevation myocardial infarction. *Postgrad Med*. Mar 2009;121(2):102-5. doi:10.3810/pgm.2009.03.1981

11.	van Kruijsdijk RC, van der Heijden JJ, Uijlings R, Otterspoor LC. Sepsis-related myocardial calcification. *Circ Heart Fail*. Sep 2011;4(5):e16-8. doi:10.1161/circheartfailure.111.962183

12.	Yoshihara S, Yaegashi T, Matsunaga M, Naito M. Rapidly progressive myocardial calcification following sepsis. *J Cardiol Cases*. Mar 2022;25(3):166-169. doi:10.1016/j.jccase.2021.08.011

13.	Caracioni AA, Mouritane S, Singh S, et al. A unique case of sepsis-triggered reverse Takotsubo cardiomyopathy resulting in cardiogenic shock. *Arch Clin Cases*. 2025;12(4):140-147. doi:10.22551/2025.49.1204.10328

14.	Garcia-Cardenas M, Jimenez-Santos M, Cabello-Ganem A, et al. Single-Photon Emission Computed Tomography Assessment of Sepsis-Related Myocardial Calcification. *Heart Lung Circ*. Apr 2023;32(4):e19-e20. doi:10.1016/j.hlc.2023.01.007

15.	Lörstad S, Tehrani S, Ekenbäck C, et al. Transient Coronary Microvascular Dysfunction in Septic Shock. *JACC Case Rep*. Jun 11 2025;30(14):103611. doi:10.1016/j.jaccas.2025.103611

16.	Paraschiv C, Nicolaescu DO, Popescu MR, et al. Laboratory and Microbiological Considerations in Sepsis-Induced Cardiac Dysfunction. *Medicina (Kaunas)*. Sep 30 2025;61(10)doi:10.3390/medicina61101765

17.	Flesch M, Kilter H, Cremers B, et al. Effects of endotoxin on human myocardial contractility involvement of nitric oxide and peroxynitrite. *J Am Coll Cardiol*. Mar 15 1999;33(4):1062-70. doi:10.1016/s0735-1097(98)00660-3
